# Tunable reporter signal production in feedback-uncoupled arsenic bioreporters

**DOI:** 10.1111/1751-7915.12031

**Published:** 2013-01-15

**Authors:** Davide Merulla, Vassily Hatzimanikatis, Jan Roelof Meer

**Affiliations:** 1 Department of Fundamental Microbiology, University of Lausanne1015, Lausanne, Switzerland; 2 Laboratory of Computational Systems Biotechnology, Ecole Polytechnique Fédérale de Lausane (EPFL)CH 1015, Lausanne, Switzerland; 3 Swiss Institute of Bioinformatics (SIB)CH 1015, Lausanne, Switzerland

## Abstract

*Escherichia coli-*based bioreporters for arsenic detection are typically based on the natural feedback loop that controls *ars* operon transcription. Feedback loops are known to show a wide range linear response to the detriment of the overall amplification of the incoming signal. While being a favourable feature in controlling arsenic detoxification for the cell, a feedback loop is not necessarily the most optimal for obtaining highest sensitivity and response in a designed cellular reporter for arsenic detection. Here we systematically explore the effects of uncoupling the topology of arsenic sensing circuitry on the developed reporter signal as a function of arsenite concentration input. A model was developed to describe relative ArsR and GFP levels in feedback and uncoupled circuitry, which was used to explore new ArsR-based synthetic circuits. The expression of *arsR* was then placed under the control of a series of constitutive promoters, which differed in promoter strength, and which could be further modulated by TetR repression. Expression of the reporter gene was maintained under the ArsR-controlled P*_ars_* promoter. ArsR expression in the systems was measured by using ArsR–mCherry fusion proteins. We find that stronger constitutive ArsR production decreases arsenite-dependent EGFP output from P*_ars_* and vice versa. This leads to a tunable series of arsenite-dependent EGFP outputs in a variety of systematically characterized circuitries. The higher expression levels and sensitivities of the response curves in the uncoupled circuits may be useful for improving field-test assays using arsenic bioreporters.

## Introduction

Bacterial bioreporters are genetically modified strains that express a reporter protein, typically a spectroscopically or electrochemically active protein, in response to a specific unique or group of related target chemicals (van der Meer and Belkin, 2010). Bioreporter assays can be a useful complement for analysis of toxic compounds in, e.g. water (Tecon *et al*., 2010) or soil samples (Paton *et al*., 2009), air (de las Heras and de Lorenzo, 2011), food-stuffs (Baumann and van der Meer, 2007), urine (Lewis *et al*., 1984) or blood serum (Turner *et al*., 2007). In certain cases where chemical analyses are too expensive or logistically difficult to perform, bioreporter assays can present an appropriate quantitative substitution. As an example, Siegfried and colleagues (2012) and Trang and colleagues (2005) successfully demonstrated large-scale and quantitative use of an *Escherichia coli*-based bioreporter assay for arsenic in drinking water from local wells in villages in Bangladesh and Vietnam respectively.

**Figure 1:**
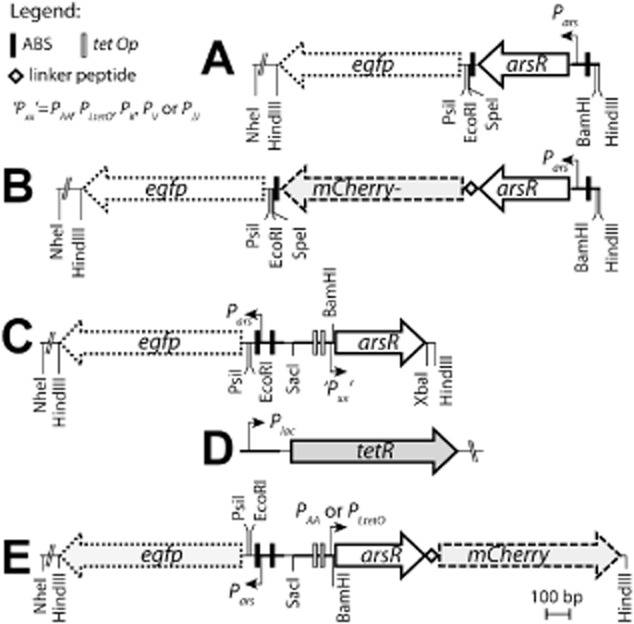
Schematic organization of the ArsR-controlled genetic circuits assembled on plasmids in *E. coli*.A. Elements building the feedback *arsR-egfp* construct.B. As (A), but with the *arsR–mCherry* fusion gene.C. The uncoupled arsenic bioreporter circuits.D. The *tetR* gene under control of the *lac* promoter. E. Uncoupled circuit with the *arsR–mCherry* fusion gene. The position of the binding site for ArsR on the DNA is depicted by dark vertical bars (ABS); those for TetR by grey vertical bars. Positions of restriction sites relevant for cloning are indicated. Outline of (C) indicative for plasmids pAAUN, pLtetOUN, pIIUN, pKUN, pVUN and pJJUN. Those in (E) for pAAUNmChe and pLtetOUNmChe.

The central element in bioreporter strains is a genetic circuit formed by the gene for a ‘sensor/transducer’ protein (e.g. a transcription regulator) and a ‘switch’ (the DNA region to which the transcription regulator binds), which controls the promoter driving expression of the reporter gene (Daunert *et al*., 2000). The DNA ‘parts’ for the genetic circuit are commonly mined from natural systems and placed in a different host cell context. Genetic circuits for arsenic detection (Ramanathan *et al*., 1997; Tauriainen *et al*., 1997; Stocker *et al*., 2003) are typically based on the bacterial arsenic defence system, like, for instance, encoded by the *arsRDABC* operon on *E. coli* plasmid R773 (Hedges and Baumberg, 1973). This system is homeostatically regulated by the ArsR and ArsD *trans*-acting repressors at the level of *ars* expression (Wu and Rosen, 1993; Bruhn *et al*., 1996; Chen and Rosen, 1997). Both ArsR and ArsD are 13 kDa protomers and form homodimers (Wu and Rosen, 1993; Rosen, 1995), but they share no sequence similarity. ArsR is an As_III_/Sb_III_-responsive repressor with high affinity for its DNA operator (named ArsR binding site or *ABS*), which is positioned upstream of the *ars* promoter (Fig. 1A) (Wu and Rosen, 1993; Rosen, 1995). ArsR binds the ABS in absence of arsenite and is thought to hinder RNA polymerase from starting transcription, thereby controlling the background expression of the *ars* operon, including of the *arsR* gene itself. Binding of arsenite or antimonite to ArsR decreases its affinity for the ABS (Wu and Rosen, 1991), and unleashes *ars* transcription. Expression of the *ars* operon is thus controlled via a feedback loop, since *arsR* is the first gene to be transcribed after derepression. ArsD is a metallochaperone that increases cellular resistance by delivering arsenite to the ArsA subunit of the extrusion system (Lin *et al*., 2006). It also controls the maximal level of expression of the *ars* operon by binding with a two orders of magnitude lower affinity than ArsR to the ABS, eventually turning *ars* expression off (Chen and Rosen, 1997). *Escherichia coli* additionally has a chromosomally encoded *ars* operon, which is formed by the *arsRBC* genes (Diorio *et al*., 1995; Chen and Rosen, 1997). ArsR^R773^ and ArsR^K12^ share 74% amino acid similarity (Fig. S1). The *ars^K12^* operon lacks *arsD* and *arsA*, an ATPase that forms a complex with the arsenite-specific membrane channel ArsB to produce the active arsenite extrusion complex (Zhou *et al*., 2000).

Most arsenic bioreporters except one (Tani *et al*., 2009) have been designed to have the reporter gene downstream of *arsR* under ArsR-feedback control of P*_ars_* (Ramanathan *et al*., 1997; Tauriainen *et al*., 1997; Stocker *et al*., 2003). When such reporter cells encounter arsenite, this will bind to the ArsR-dimer, causing it to dissociate from its binding site and unleashing further expression of itself and of the reporter gene. The increase in reporter protein expression and activity is approximately linear in the range between 5 and 80 μg of arsenite per litre (Stocker *et al*., 2003; Baumann and van der Meer, 2007), and can be used to quantify arsenite concentrations in unknown samples. However, since the feedback loop is essentially a bit leaky to allow formation of ArsR that needs to repress the system, background reporter gene expression in the absence of arsenite may be disturbingly high (Stocker *et al*., 2003). In a conceptually very different reporter circuit configuration, expression of *arsR* is uncoupled from its feedback loop, whereas the reporter gene expression is maintained under ArsR control via the P*_ars_* promoter and the ABS (Fig. 1C). In this case an arsenite-independent promoter controls the expression of *arsR* such that ArsR levels are sufficient to repress the background expression of the reporter gene from the P*_ars_* promoter are constitutively produced.

The objectives of the current work were to systematically explore the effects of arsenite concentration-dependent reporter gene expression in the uncoupled circuitry mode. A mechanistic model was developed for ArsR repression of P*_ars_* based on mass action kinetics, analogous to a model for LacI repression of P*_lac_* (Lee and Bailey, 1984) to predict the effects of feedback and uncoupled circuitry on ArsR and EGFP expression. The model was tested experimentally by varying ArsR concentrations over a wide range using two promoters with different maximal strength that were placed under control of TetR and could be derepressed by addition of anhydrotetracycline (aTc). In order to estimate relative changes in intracellular ArsR concentrations we used additional gene circuitry with *arsR–mCherry* fusions instead of *arsR* (Fig. 1B and E). Since pre-induction with aTc is not practical in field assays, we then replaced TetR-regulatable expression by a set of constitutive promoters with different (published) strengths (Alper *et al*., 2005) (Fig. S2), and tested the EGFP output as a function of arsenite concentrations in *E. coli* strains with or without chromosomal *arsRBC* gene cassette. We find that uncoupling can have important gain on reporter output and can result in modulatable maximum reporter levels.

## Results

### Uncoupling *arsR* expression is predicted to produce tunable reporter signal development

**Figure 2:**
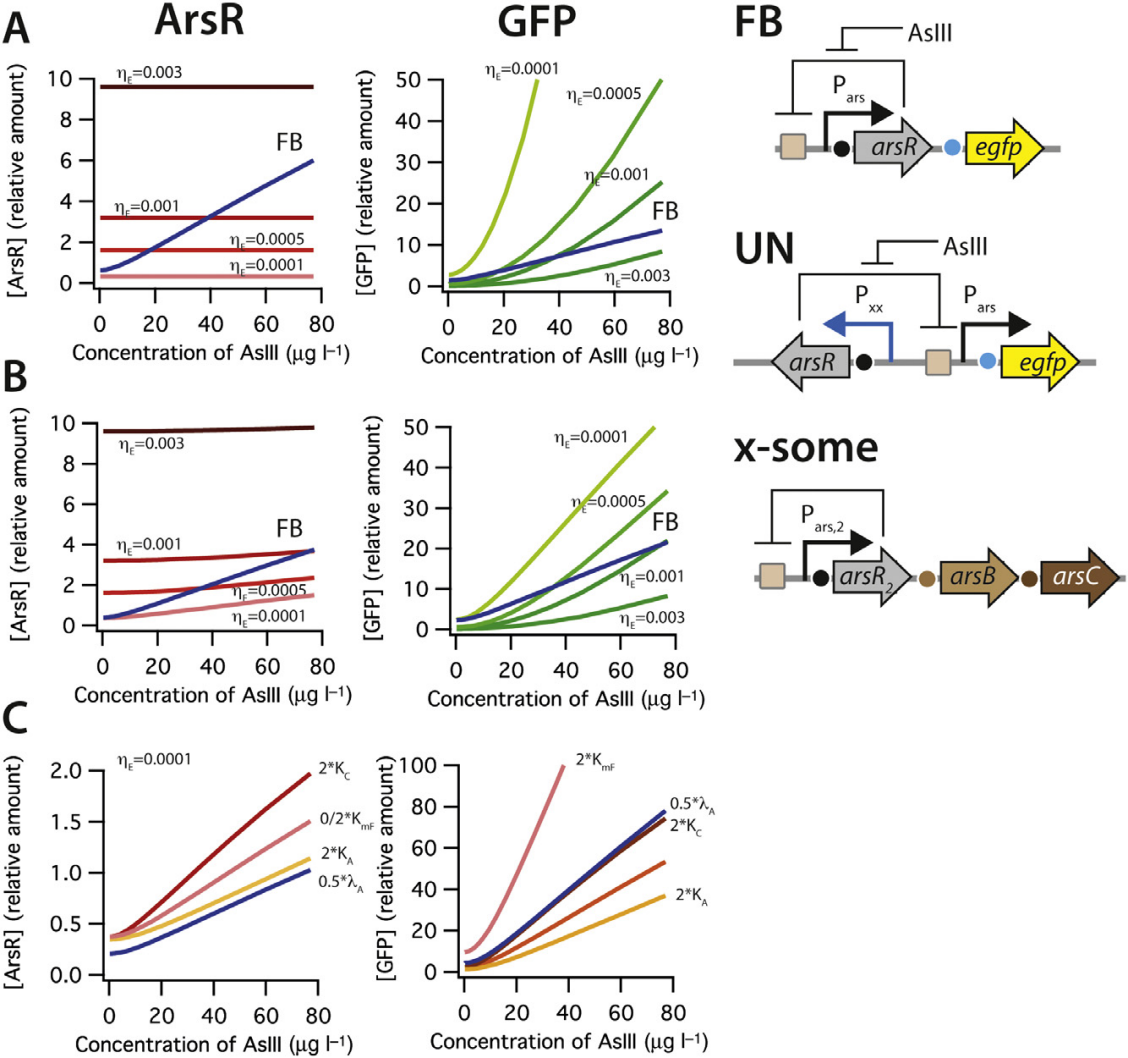
A and B. Predictions of ArsR and EGFP mass action equilibrium concentrations as a function of arsenite (AsIII) exposure for the original plasmid feedback circuit (FB), for the plasmid uncoupled circuit (UN), in absence (A) or presence (B) of an extra chromosomal *arsR* (x-some). Parameters: η_E_, transcription efficiency of the constitutive promoter for *arsR* (P*_xx_*).C. Sensitivity analysis on case (B) with η_E_ = 0.0001 varying K_mF_, EGFP translation efficiency; K_C_, equilibrium constant of ArsR for AsIII; K_A_, equilibrium constant of ArsR with its DNA binding site; λ_A_, number of ArsR protein per *arsR* mRNA. For details of model assumptions, see *Supporting information* and SI File 1.

The behaviour of the ArsR-P*_ars_* feedback (FB) system can be predicted using a mechanistic model based on mass action binding equilibria between ArsR and its DNA binding sites, ArsR and arsenite, and RNA polymerase and the *arsR* promoter, analogous to a model described for LacI control of the *lac* promoter (Lee and Bailey, 1984) (*Supporting information*). The predicted relative concentrations of ArsR and EGFP produced under steady-state conditions as a function of exposure to arsenite both increase over the range of 0–80 μg of AsIII per litre (Fig. 2A, *FB*), for a situation with *arsR* present only on a plasmid in the cell. Note that we consider here only the typical measurement range of arsenite concentrations for the arsenic bioreporter. The model in Supporting information (SI) File 1 allows interested readers to test other concentration ranges. In case of an additional chromosomal *arsR* copy, the arsenite-dependent production of ArsR would be slightly lower and that of EGFP slightly higher (Fig. 2B, FB). We next examined the model prediction for the case where expression of ArsR is ‘uncoupled’ from its feedback control, whereas that of EGFP is maintained under arsenite-dependent ArsR/P*_ars_* control. In this scenario *arsR* transcription can be varied by using different strength promoters, or giving different transcription efficiencies (η_E_ in the model). Accordingly, the model predicts that by varying the promoter strength for *arsR* expression across a 30-fold range (η_E_ in Fig. 2A and B) one could achieve ArsR levels in the cell that are constantly lower (η_E_ = 0.0001) or higher (η_E_ = 0.003) than in the feedback system. Interestingly, maintaining constant ArsR production at different levels is predicted to result in largely different response curves of the EGFP signal produced from P*_ars_*. Higher ArsR levels (e.g. η_E_ = 0.003) will lead to less steep EGFP response curves as a function of arsenite exposure, whereas lower levels (η_E_ = 0.0001) are predicted to lead to steeper response curves (Fig. 2A). Noteworthy, predictions suggest that maintaining a chromosomal *arsR* copy would result in slightly lower EGFP outputs for the case of the uncoupled gene circuitry. It is important to further note that the model is not a data ‘fitting’ but a mechanistic model, allowing to systematically explore variations in underlying parameters. As an example, the model predicts the reporter output to be relatively sensitive to changes in the equilibrium binding constant of ArsR with AsIII (K_C_, Fig. 2C).

### Tunable uncoupling effects on EGFP expression

**Figure 3:**
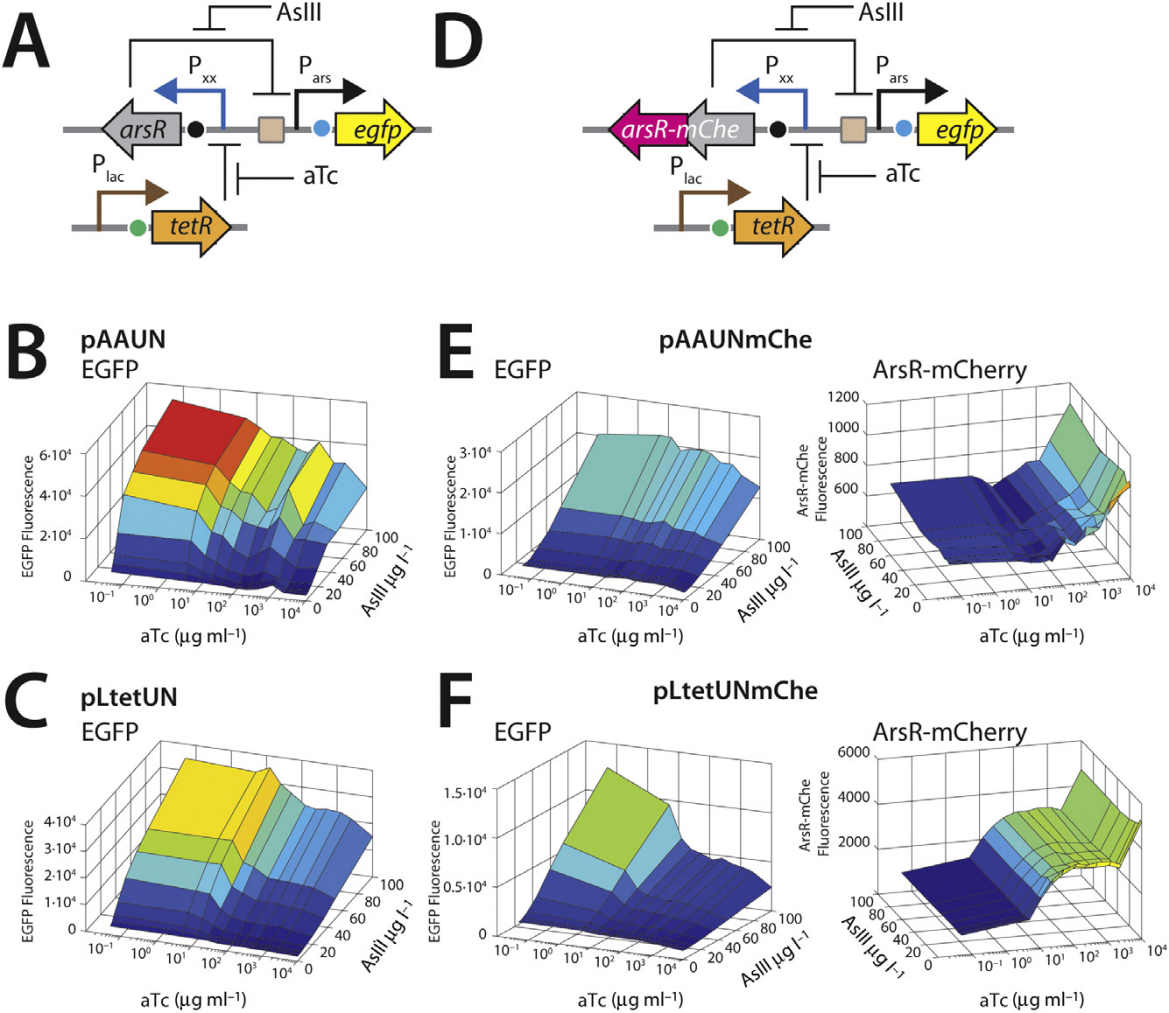
Systematic effects of varying ArsR production on the arsenite-dependent EGFP synthesis from P*_ars_* in *E. coli* MG1655 ΔRBC.A–C. (A) Relevant uncoupled circuitry design with P*_xx_* being either P*_AA_* (B) or P*_LtetO_* (C).D–F. Relevant uncoupled circuitry design for the *arsR–mCherry* fusion variant circuitry with P*_xx_* being either P*_AA_* (E) or P*_LtetO_* (F). Notice log scale for aTc addition and different scale for EGFP or ArsR–mCherry fluorescence between panels. Fluorescence measured by flow cytometry on cells pre-induced for 2 h with aTc, and subsequently 3 h with arsenite.

To experimentally explore and verify the predicted effects of uncoupling the ArsR-P*_ars_* feedback loop on reporter gene induction, we constructed a series of new topologies in which *arsR* expression is controlled from a defined promoter, whereas ArsR still controls the expression of the reporter gene (*egfp*) via P*_ars_* (Fig. 2, UN). Since the native P*_ars_* expression feedback loop has a relatively high background expression, we used a variant in which a second ArsR binding site is inserted downstream of *arsR* in the feedback circuit, which reduces background expression in the absence of arsenite (Stocker *et al*., 2003). This secondary ArsR binding site is maintained in the uncoupled versions (Fig. 1). Furthermore, to experimentally create the condition of having only a plasmid-located *arsR* gene circuit we deleted the chromosomal *arsRBC* cassette in *E. coli* MG1655. Tunable expression of *arsR* was achieved by using two constitutive promoters (P*_LtetO_* and P*_AA_*) that have additional TetR recognition sites within their promoters (Figs S2 and S3). Expression of *arsR* can then be brought under control of TetR by including a P*_lac_*-expressed *tetR* gene on a secondary plasmid (Figs 1 and 3A). The output of the P*_LtetO_* and P*_AA_* promoters was systematically increased by pre-incubation with defined aTc concentrations for 2 h, after which the cells were exposed to arsenite to follow reporter induction from P*_ars_*. Increasing the aTc concentration will on average lead to more derepression of TetR control on ArsR, as a result of which more ArsR is produced that can repress the P*_ars_* promoter. The consequence of this is a less steep EGFP reporter curve (Fig. 3B and C). In the absence of aTc repression by TetR is maximal, causing minimal ArsR production and highest arsenite-dependent EGFP expression. At the highest aTc concentration ArsR levels were maximal and arsenite-dependent production of EGFP was minimal, which is conform the model predictions. One can observe that the P*_LtetO_* promoter is indeed stronger than P*_AA_* since the EGFP response curve is lower at the highest aTc concentrations. Interestingly, both model predictions and experimental data confirm that even the strongest promoter for *arsR* expression will not completely abolish arsenite-dependent expression from P*_ars_* (Figs 2 and 3).

To demonstrate that indeed higher ArsR levels are responsible for this behaviour we produced variant reporter circuits in which the *arsR* gene is fused via a short linker to *mCherry*, which leads to an ArsR–mCherry fusion protein (Fig. 3D). Comparatively, the circuits with the ArsR–mCherry fusion protein produced only half the EGFP reporter output as those with ArsR (Fig. 3E and F). This indicated that ArsR–mCherry is still functional, but the model predicts that it must have a stronger binding constant to the ArsR binding site, since EGFP production is lower than for the ArsR system at the same arsenite concentration (Fig. 2C, stronger binding constant would be equivalent to changing the value for K_A_. Compare EGFP responses for K_A_ and 2×K_A_). As expected from the model predictions the amount of ArsR–mCherry protein, taken as the intensity of mCherry fluorescence, increased with increasing aTc concentration in the pre-incubation step, was independent of the arsenite concentration, and was higher for the P*_LtetO_* -driven than the P*_AA_*-driven system (Fig. 3E and F).

### Uncoupling effects in modular strains with different constitutive *arsR* control

**Figure 4:**
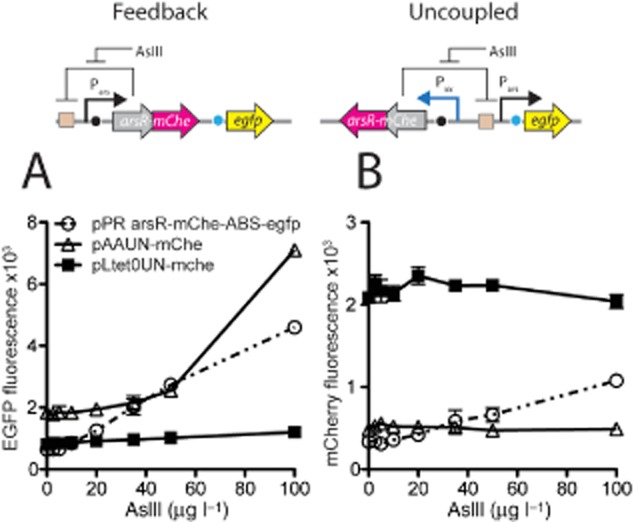
Effects of uncoupled versus feedback circuit in the absence of TetR control in *E. coli* MG1655 ΔRBC.A. EGFP fluorescence as a function of arsenite exposure, measured 180 min after induction using flow cytometry.B. mCherry fluorescence from ArsR–mCherry as a function of arsenite exposure, in the same cells as in (A).

Because pre-induction with aTc is not a practical solution for a bioassay we tested the same circuits in a background without *tetR* but varying only the promoter strength for *arsR*. Indeed, we observed that the levels of ArsR-mCherry were independent of the arsenite concentration in the strains with the uncoupled circuits (Fig. 4B), whereas those in the strain with the feedback circuit increased with increasing arsenite concentration. As expected from the model the circuit with the stronger promoter for *arsR–mCherry* (P*_LtetO_*) produced more ArsR–mCherry but less EGFP output than the circuit with the weaker P*_AA_* promoter (Fig. 4A). In contrast, but also according to model predictions, the background EGFP expression in absence of arsenite was higher in the uncoupled circuit with the weaker promoter.

**Figure 5:**
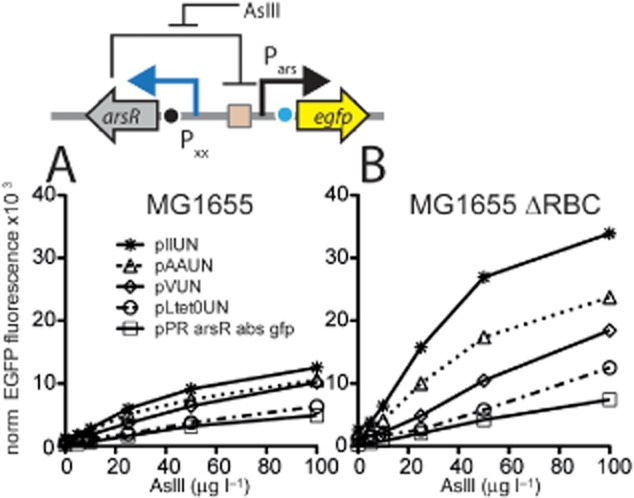
Arsenite-dependent EGFP fluorescence in cultures of *E. coli* MG1655 (A), and *E. coli* MG1655 ΔRBC (without the chromosomal *arsRBC* cassette; B) carrying the original feedback circuit (pPR-arsR-ABS) or four uncoupled *arsR* reporter circuits with different promoter strengths driving *arsR* expression (pAAUN, pLtetOUN, pIIUN, pVUN). Fluorescence measured by flow cytometry after 180 min induction time. Data symbols represent the average from independent biological triplicates. Whiskers, SD (when not visible lay within the symbol size).

Because these circuits were tested with the ArsR–mCherry variant, which had a stronger repression effect than native ArsR, we finally replaced the native *arsR* gene back instead of *arsR–mCherry* under control of variant constitutive promoters with different (published) strengths, from the weakest P*_II_* to the strongest P*_LtetO_* (Alper *et al*., 2005) (Table 1, Fig. S2). Results showed a range of EGFP outputs with increasing fluorescence for the same arsenite exposure concentration at weaker promoter strengths for *arsR* expression. The weakest promoter for *arsR* in the construct pIIUN resulted in up to fivefold higher EGFP fluorescence than in the original feedback construct pPR-ArsR-ABS at the same arsenite concentration (Fig. 5B). Interestingly, and stronger than expected from the model, the EGFP output of the same circuit in *E. coli* without the chromosomal *arsRBC* cassette (MG1655ΔRBC) was more than twice as strong as in wild-type *E. coli* MG1655 (Fig. 5A). The reason for this may be that because the *arsR* chromosomal copy is not completely identical to the plasmid *arsR* copy, their mutual repression is different than the model assumes for reasons of simplicity. Incidentally, measuring the induction from the same reporter circuits by fluorometry produces approximately similar response curves (Fig. S4). Kinetic profiles of reporter gene induction under the used assay conditions all show a typical 40 min lag during which hardly any increase of reporter signal is observed (Fig. S5).

**Table 1:** Used strains and plasmids in this study

Strain number	Host strain	Relevant genotype	Plasmid	Reference
1598	*Escherichia coli* DH5α	KmR	pPR-arsR-ABS-egfp	Stocker *et al*. (2003)
3391	*E. coli* MG1655 ΔRBC	Deletion of *arsRBC*, KmR	pAAUN	This study
3316	*E. coli* MG1655 ΔRBC	Deletion of *arsRBC*, KmR	pPR-arsR-ABS-egfp	This study
3304	*E. coli* MG1655 ΔRBC	Deletion of *arsRBC*	–	This study
3328	*E. coli* MG1655	Wild-type, KmR	pPR-arsR-ABS-egfp	This study
3307	*E. coli* MG1655	KmR	pAAUN	This study
3612	*E. coli* MG1655	KmR	pJJUN	This study
3633	*E. coli* MG1655	KmR, ArsR–mCherry fusion	pJJUN-mChe	This study
3636	*E. coli* MG1655	KmR	pKUN	This study
3614	*E. coli* MG1655	KmR	pLtetOUN	This study
3634	*E. coli* MG1655	KmR, ArsR–mCherry fusion	pLtetOUN-mChe	This study
3652	*E. coli* MG1655 ΔRBC	Deletion of *arsRBC*, KmR	pLtetOUN	This study
3653	*E. coli* DH5α	KmR	pLtetOUN	This study
3660	*E. coli* MG1655 ΔRBC	Deletion of *arsRBC*, KmR, ArsR–mCherry fusion	pLtetOUN-mCherry	This study
3665	*E. coli* DH5α	KmR	pAAUN	This study
3668	*E. coli* DH5α	KmR, ArsR–mCherry fusion	pAAUN-mChe	This study
3670	*E. coli* MG1655	KmR	pVUN	This study
3792	*E. coli* DH5α	KmR, ArsR–mCherry fusion	pPR-arsR-mChe-ABS-egfp	This study
3795	*E. coli* MG1655 ΔRBC	Deletion of *arsRBC*, KmR, ArsR–mCherry fusion	pPR-arsR-mChe-ABS-egfp -	This study
4210	*E. coli* MG1655 ΔRBC	Deletion of *arsRBC*, KmR, ApR, ArsR–mCherry fusion, P*_lac_* driven TetR expression	pLtetOUN-mCherry/pGem-TetR	This study
4222	*E. coli* MG1655 ΔRBC	Deletion of *arsRBC*, KmR, ApR, ArsR–mCherry fusion, P*_lac_* driven TetR expression	pAAOUN-mCherry/pGem-TetR	This study

ApR, ampicillin resistance; KmR, kanamycin resistance.

### Cell to cell variation in reporter expression in feedback versus uncoupled circuits

**Figure 6:**
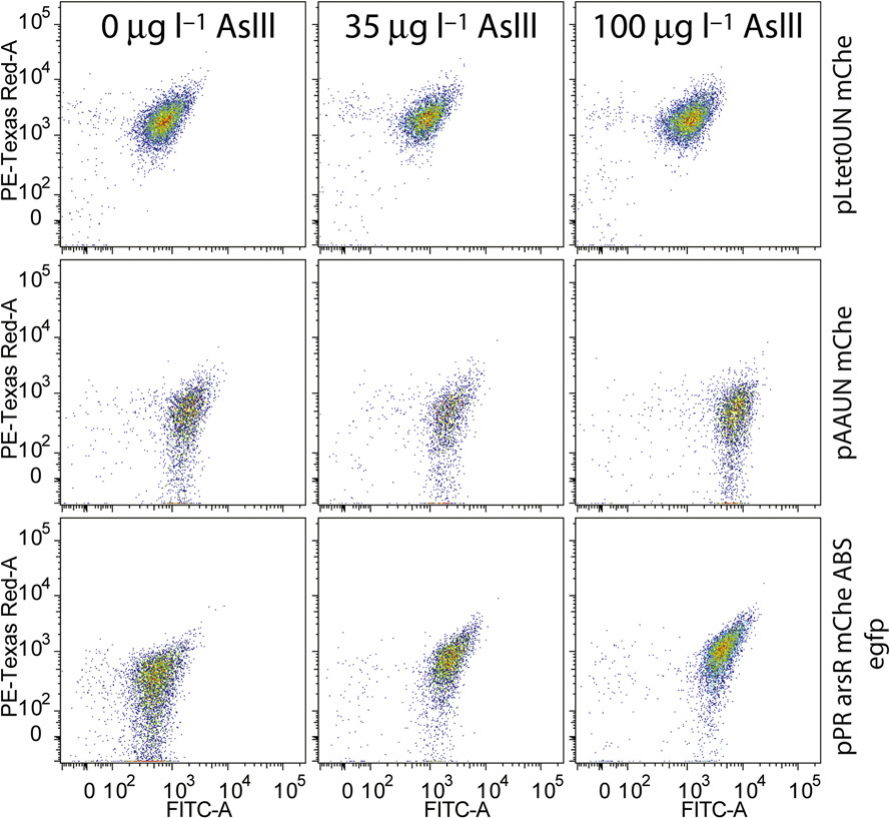
FC analysis of single-cell heterogeneity of ArsR–mCherry and EGFP expression in *E. coli* MG1655 ΔRBC coupled and uncoupled bioreporters after 3 h exposure to arsenite at the indicated concentrations. Dot plots show EGFP (FITC-A channel) versus mCherry fluorescence (PE-Texas Red-A channel) of single cells on a ^10^log-scale for ∼ 2000 events per sample.

EGFP expression heterogeneity among individual cells (expressed as the mean SD from the FC FITC channel distributions) was significantly smaller (∼ 1.4-fold, when expressed as ratio between the SD normalized as percentage of the respective means) for cells in the presence of arsenite than without, but only in the case of the feedback circuit (Fig. 6, pPR-arsR-mChe-ABS-egfp; Table 2). Single-cell EGFP and mCherry fluorescence correlated positively for the feedback but not for the uncoupled circuit (Table 2). Normalized SD of both EGFP and mCherry among individual cells were lower for the stronger promoter in the uncoupled circuit (pLtetOUN, Fig. 6).

**Table 2:** Analysis of reporter protein variation in feedback versus uncoupled ArsR-controlled circuits in *E. coli* MG1655 ΔRBC

Circuit	Arsenite concentration (μg l^−1^)	Average EGFP^a^	Average SD^b^	SD per cent of average^c^	Average mCherry^a^	Average SD mCherry^b^	SD per cent of average^c^	Pearson correlation factor (*r*)^d^
pPR-arsR-mChe-ABS	0	605 ± 18	504 ± 136	83	345 ± 18	345 ± 18	98	0.5216
	2.5	646 ± 33	477 ± 66	74	339 ± 26	364 ± 11	107	0.4221
	5	655 ± 33	471 ± 19	72	311 ± 9	375 ± 35	121	0.6183
	10	843 ± 65	536 ± 13	64	357 ± 17	381 ± 18	107	0.5805
	20	1253 ± 50	733 ± 7	58	419 ± 8	398 ± 18	95	0.6181
	35	2028 ± 257	1169 ± 102	58	584 ± 136	495 ± 85	85	0.7143
	50	2733 ± 123	1504 ± 98	55	661 ± 83	543 ± 73	82	0.7075
	100	4601 ± 47	2295 ± 90	50	1077 ± 22	708 ± 63	66	0.748
pAAUN-mChe	0	1836 ± 24	1095 ± 50	60	483 ± 12	485 ± 29	100	0.4986
	2.5	1779 ± 113	1207 ± 13	68	523 ± 9	558 ± 22	107	0.5924
	5	1836 ± 208	1243 ± 117	68	554 ± 44	569 ± 51	103	0.5777
	10	1835 ± 133	1305 ± 32	71	518 ± 3	533 ± 26	103	0.5352
	20	1942 ± 172	1396 ± 48	72	515 ± 26	557 ± 48	108	0.6204
	35	2146 ± 231	1516 ± 233	71	507 ± 56	549 ± 30	108	0.5626
	50	2549 ± 199	1753 ± 61	69	470 ± 7	501 ± 31	107	0.5063
	100	7101 ± 129	3712 ± 85	52	489 ± 12	500 ± 56	102	0.3341
pLtet0UN-mChe	0	799 ± 14	424 ± 12	53	2084 ± 44	1320 ± 83	63	0.6042
	2.5	851 ± 17	463 ± 12	54	2261 ± 102	1446 ± 151	64	0.5457
	5	845 ± 25	457 ± 15	54	2165 ± 137	1862 ± 746	86	0.5623
	10	868 ± 7	479 ± 9	55	2136 ± 92	1343 ± 33	63	0.5299
	20	912 ± 8	500 ± 24	55	2351 ± 107	1653 ± 182	70	0.5866
	35	956 ± 6	511 ± 6	53	2231 ± 67	1361 ± 53	61	0.5414
	50	1016 ± 25	550 ± 36	54	2234 ± 65	1310 ± 56	59	0.4568
	100	1199 ± 17	638 ± 13	53	2037 ± 81	1504 ± 455	74	0.497

a Averages from three independent replicates ± one calculated standard deviation (SD) on the average. Signals averaged from 10 000 events per replicate.

b Average of SD calculated from 10 000 events per replicate ± one calculated SD on the average. This average is an indication for the variation of reporter expression among single cells in the population.

c Percentage of the average SD of the total average EGFP or mCherry.

d Correlation between EGFP and mCherry signals of each single cell.

## Discussion

We focused in this work on a systematic analysis of the effects on reporter gene expression from the *ars* promoter when decoupling synthesis of ArsR itself from its regular feedback loop. Controlling the expression of the circuit regulator by synthetic constitutive rather than cognate promoters has been shown previously to improve reporter output (Wu *et al*., 2009) but this has not been tested very systematically. As a first research question we examined whether the level of constitutive expression of ArsR would influence the reporter output from P*_ars_*. A mechanistic model was developed for ArsR-dependent EGFP expression from P*_ars_*, which non-intuitively predicted that constitutive promoters with a 30-fold different ‘strength’ would largely change the output of the circuitry in response to arsenite (Fig. 2). Experimental verification using TetR-aTc modulatable expression of *arsR* confirmed the model predictions to a large extent, except for details in the background expression level in absence of arsenite. Although the TetR-aTc system can be used for stepwise modulation of ArsR production, the pre-incubation with aTc is not very practical in a field assay. We then therefore replaced the TetR-aTc by a subset of constitutive promoters of different strength, which had been derived from P*_LtetO_* (Lutz and Bujard, 1997) by random mutagenesis (Alper *et al*., 2005) (Table 1, Fig. S2). Based upon the relative amount of mRNA produced from the specific promoter compared with the amount produced from P*_LtetO_* (= 1), Alper and colleagues (2005) ranked the promoters as 0.57 for P*_V_*, 0.30 for P*_K_*, 0.24 for P*_AA_*, 0.16 for P*_JJ_* and 0.06 for P*_II_*. By deducing from the fluorescence light intensity of ArsR–mCherry fusion proteins in single cells (FC) we could confirm that P*_LtetO_* was the stronger promoter than P*_AA_* (Figs 3 and 4). This experiment showed directly that higher ArsR–mCherry production leads to a reduction of the formation of EGFP from P*_ars_* as a function of arsenite exposure (Fig. 4).

Second, the model also predicted that different strength constitutive promoters for *arsR* expression would influence the shape of the arsenite-dependent response curve of the reporter protein (Fig. 2). Experiments with all six promoters confirmed that the amount of EGFP reporter signal produced from P*_ars_* as a function of arsenite exposure can be tuned by the strength of the promoter controlling the transcription of *arsR* (Fig. 5). Interestingly, these results also demonstrated that even the strong P*_LtetO_* promoter is insufficient to produce ArsR to such a level as to completely repress P*_ars_* in presence of arsenite (Figs 2 and 5). In contrast, the ArsR–mCherry fusion protein produced from P*_LtetO_* (Fig. 4A) was sufficient to completely repress P*_ars_*, which suggest that although ArsR–mCherry is functional and responsive to arsenite, its stability or DNA binding properties are enhanced and, consequently, its repression of the *ars* promoter is more severe.

Single-cell analysis of the reporter responses in the feedback (pPR-ArsR-mChe-ABS-egfp) and uncoupled system (pAAUN-mChe, pLTet0UN-mChe) showed that the cells with the feedback system tend to have larger variation in EGFP and mCherry produced from P*_ars_* at low arsenite concentrations, which successively become smaller at higher arsenite concentrations (Table 2). EGFP and ArsR–mCherry fluorescence in those cells correlate positively at higher arsenite exposures, meaning that cells which accidentally have higher ArsR–mCherry levels also (have) produce(d) more EGFP. This is conform the model predictions in Fig. 2 but counterintuitive for the supposed negative feedback exerted by ArsR, which would dictate that (temporarily) higher intracellular ArsR concentrations would tend to suppress the P*_ars_* promoter. In that case, there would not be a correlation between ArsR–mCherry and EGFP levels in the same cell. While keeping in mind that ArsR–mCherry does not behave exactly as ArsR itself our observations thus suggests either that there are oscillations in P*_ars_* expression at single-cell level which we cannot detect because of using stable EGFP, or that a part of the produced ArsR–mCherry is not engaged in binding its promoter (e.g. by being permanently bound to arsenite). Modelling and experimental measures of GFP output from an engineered *lacI*-based negative feedback circuit showed that single feedback circuits can indeed produce reporter oscillations, although not as pronounced as typical oscillatory double loop genetic circuits (Stricker *et al*., 2008). This may be further explored for the ArsR-controlled circuits by expanding the mechanistic model presented here to a stochastic single-cell model. The EGFP reporter variation per cell in the uncoupled circuits depends on the strength of the promoter used to produce ArsR–mCherry, and diminishes at higher ArsR–mCherry levels. However, in contrast to the feedback circuit, variation in EGFP expression for the uncoupled circuits across single cells in a population does not diminish at higher arsenite concentrations (Table 2). Also, there is a poorer correlation for the uncoupled circuits between the ArsR–mCherry level in single cells and their EGFP level (Table 2), meaning that cells can have considerable variation in ArsR–mCherry but still produce the same amount of EGFP from P*_ars_*. Understanding and controlling single-cell variation in reporter gene expression may be useful for more assays that capture the responses of relatively few cells such as, e.g. in microfluidics systems (Buffi *et al*., 2011).

In summary, the results of the presented work show how the P*_ars_*-*arsR* feedback loop can be uncoupled to produce a tunable expression system with the advantage of increasing the linear operational range or intensity of the response. The higher reporter outputs may be useful for improving the detection range in, e.g. field test assays focusing on measuring arsenic in potable water sources (Trang *et al*., 2005; Siegfried *et al*., 2012). Better understanding of the ArsR-feedback circuit may also provide alternative models for genetic circuitry, which typically concentrate on a limited number of inducible or repressible systems with little relevance for environmentally useful bioreporters.

## Experimental procedures

### Strains and culture conditions

All strains, plasmids and relevant characteristics are listed in Table 1. *Escherichia coli* strains were generally cultured on LB medium (Sambrook and Russell, 2001) at 37°C with inclusion of the appropriate antibiotics to maintain the plasmid reporter constructs, as indicated in Table 1.

### Design of the arsenic reporter circuits

In the new *ars* reporter circuits the expression of *arsR* is uncoupled from its own natural P*_ars_* promoter, whereas the reporter gene remains under ArsR-repressible P*_ars_* control (Fig. 1). A synthetic DNA fragment was produced (DNA2.0, Menlo Park, CA, USA) containing *arsR* positioned under the control of the weak P*_AA_* constitutive promoter described by Alper and colleagues (2005), fused to a divergently oriented *P_ars_* promoter and a second ArsR binding site (ABS, Fig. 1). This 688 bp fragment (*ABS_P_ars__P_AA__arsR*) further contains specific unique restriction sites by which each individual element is interchangeable (Fig. 1). The fragment was cloned in front of the *egfp* reporter gene of pPROBE-tagless (Miller *et al*., 2000) using EcoRI and XbaI digestion. After ligation and transformation into *E. coli* DH5α this resulted in plasmid pAAUN. pLtetOUN, pVUN, pIIUN, pJJUN and pKUN derive from pAAUN by substituting P*_AA_* with the resynthesized P*_LtetO_*, P*_V_*, P*_II_*, P*_JJ_* or P*_K_* promoter fragments (Alper *et al*., 2005) (DNA2.0) via cloning in the unique SacI and BamHI sites. The integrity of the new assemblies on both plasmids was verified by DNA sequencing. The relevant part of the DNA sequence characteristic for this new family of constructs with all the different promoter regions is presented in Fig. S2 (*Supporting information*). An *arsR–mCherry* fusion was created by using a previously developed plasmid encoding a variant mCherry with a 15-amino-acid linker at its N-terminal end (Miyazaki *et al*., 2012). Plasmids pAAUN-mChe and pLtetOUN-mChe resulted from cloning the linker-*mCherry* fragment in plasmids pAAUN or pLtetOUN using the HindIII site. Proper insertions were validated by DNA sequencing (Fig. 1C and D). All the primers used for sequence verification are listed in Table S1 of *Supporting information*. An equivalent variant of pPR-ArsR-ABS was constructed by resynthesizing an *arsR–mCherry* gene fragment (DNA2.0) with the appropriate restriction sites (BamHI and SpeI) and replacing the *arsR*-gene in pPR-ArsR-ABS with the *arsR–mCherry* fusion (Fig. 1B). *TetR* was amplified from pME6012 (Heeb *et al*., 2000) in the PCR with specific primers (Table S1), and cloned in pGEM-T-Easy (Promega) downstream of P*_lac_* to produce pGem-TetR. The correct direction of the insertion was determined by sequencing on the resulting plasmids. Plasmid pGem-TetR was then introduced into *E. coli* MG1655 ΔRBC carrying either pAAUN or pLtet0UN.

### Construction of chromosomal *ars* gene deletion

In order to test the influence of the native chromosomal *ars* operon on the functioning of the arsenic reporter constructs, we deleted the complete (Δ*arsRBC*) *ars* operon of *E. coli* MG1655. This was accomplished using a modification of the I-*Sce*I recombination–digestion system (Martinez-Garcia and de Lorenzo, 2011). This system is composed of a suicide plasmid pJP5603-IsceIv2, containing a kanamycin resistance cassette and a site for the intron-specific restriction enzyme I-*Sce*I, on each side of which two regions identical to the areas flanking the chromosomal fragment to be deleted can be cloned. For the complete *ars* operon deletion this consisted of fragments upstream of *arsR* and downstream of *arsC* (pJP5603-SceIv2ExtRC). Up- and downstream fragments were amplified by PCR using primers listed in Table S1, then cloned into pGEM-T-Easy and verified for correctness by DNA sequencing. Subsequently, they were retrieved by restriction digestion and cloned into pJP5603-ISceIv2. Appropriate purified pJP5603-derived plasmids were transformed into *E. coli* MG1655 and single recombinants were selected for kanamycin resistance. Recombination was verified by PCR amplification and when correct, those strains were subsequently transformed with the second plasmid pSW(ISceI), which carries an ampicillin resistance and bears the gene for I-*Sce*I under the control of the P*_m_ m*-toluate-inducible promoter (Martinez-Garcia and de Lorenzo, 2011). Transformants were selected by ampicillin resistance and then induced for production of I-*Sce*I by adding *m-*toluate at 15 mM. Ampicillin-resistant but kanamycin-sensitive colonies were subsequently screened by PCR for the absence of the targeted chromosomal region or for reversion to wild-type (Table S1). In case of correct deletions the strains were grown in multiple batch passages on LB medium without ampicillin until they were cured from the pSW(ISceI) plasmid.

### Bioreporter cultivation

Starting from a single colony, the bioreporter strain was grown for 16 h at 37°C in LB medium in the presence of 50 μg ml^−1^ kanamycin to select for the presence of the pPROBE-based reporter plasmid and, when required, 100 μg ml^−1^ ampicillin to select for pGem-TetR, with 160 r.p.m. agitation of the culture flask. The bacterial culture was then 100-fold diluted into fresh LB medium plus kanamycin and incubated for 2 h at 160 r.p.m. agitation until the culture turbidity at 600 nm had reached between 0.3 and 0.4 for the flow cytometry (FC) assay, and between 0.4 and 0.7 for the fluorimeter assay (representative for mid-exponential-phase cells). When pre-incubation with anhydrotetracycline (aTc) was required the bacterial culture was 50-fold diluted in 5 ml of LB media supplemented with kanamycin, ampicillin and 50 μl of stock solutions of aTc ranging between 0 and 1 mg per millilitre, prepared by dissolution and successive serial dilutions in HPLC degree ethanol of pure anhydrotetracycline (IBA, Göettingen).

Cells from 10 ml of culture, or 5 ml in case of aTc pre-incubation, were harvested by centrifugation at 4000 *g* for 5 min and at room temperature. The cell pellet was resuspended into 30°C preheated MOPS medium to a final optical density at 600 nm of 0.4 for the fluorimeter assay and 0.2 for the FC assay [MOPS medium contains 10% (v/v) of MOPS buffer, 2 mM MgCl_2_, 0.1 mM CaCl_2_, 2 g of glucose per litre, and is set at pH 7.0]. MOPS buffer itself was prepared by dissolving, per litre: 5 g of NaCl, 10 g of NH_4_Cl, 98.4 g of 3-([N-morpholino]propanesulfonic acid, sodium salt), 0.59 g of Na_2_HPO_4_·2H_2_O and 0.45 g of KH_2_PO_4_.

### Bioreporter assay preparation and readout

Both fluorimeter and FC bioreporter assays were prepared in triplicates in 96-well microplates (Greiner μCLEAR-BLACK). An aliquot of 180 μl of bioreporter suspension was mixed with 20 μl of aqueous solution containing between 0 and 1000 μg of arsenite (As_III_) per litre, prepared by serial dilution of a 0.05 M solution of NaAsO_2_ (Merck) in arsenic-free tap water. Bioreporter assays for fluorometry were incubated at 30°C and were mixed at 500 r.p.m. for 30 s every 10 min using a multiplate reader (FLUOstar Omega, BMG LABTECH), after which EGFP fluorescence (at 480 nm excitation and 520 nm collection) and culture turbidity (at 600 nm) were measured automatically. Reported EGFP and mCherry fluorescence values from fluorometry were normalized for culture turbidity (NFU). Bioreporter assays measured by FC were incubated at 30°C and were mixed at 500 r.p.m. for 3 h in a 96-well thermostated shaker (THERMOstar-BMG Labtech). After incubation 5 μl of all samples were diluted twice in 195 μl of distilled water, and 3 μl volume of each triplicate was aspired and analysed on a Becton Dickinson LSR-Fortessa (BD Biosciences, Erembodegem, Belgium). mCherry fluorescence of individual cells was collected in the ‘Texas-Red’ channel (610/20 nm), whereas EGFP fluorescence was registered in the ‘FITC’ channel (530/30 nm). FC fluorescence values were reported as such and not further normalized.

### Modelling the ArsR-P_ars_ system in the feedback and uncoupled configurations

A mechanistic model was developed for ArsR-mediated control of the P*_ars_* promoter using equilibrium binding affinities, in analogy of a LacI-P*_lac_* model developed by Lee and Bailey (Lee and Bailey, 1984). This model can be solved algebraically under equilibrium conditions and allows to express formation of ArsR and EGFP as a function of arsenite concentration. Essentially four configurations were modelled: (1) *arsR* and *egfp* under control of P*_ars_* (Feedback), but only plasmid copies, (2) as (1), but including a chromosomal copy of *arsR* and P*_ars_*, (3) *arsR* expression under control of a constitutive promoter with defined strength, *egfp* expression under control of P*_ars_* (uncoupled), but only plasmid copies, and (4) as (3), but including a chromosomal copy of *arsR* and P*_ars_*. Details of the model descriptions, mathematical functions and parameters are presented in *Supporting information*. An Excel version of the model is included as SI File 1, by which interested readers can vary model parameters or arsenite concentration ranges.

## Conflict of interest

None declared.

## Supporting information

**Mathematical model for ArsR circuits.**

**Table S1.** List of all the primers used in the present work showing sequence, length and melting temperature (Tm). **Fig. S1.** Nucleotide alignment of the *arsR^R73^* and the chromosomal *arsR^K12^* genes. **Fig. S2.** Relevant part of the DNA sequence of the different promoters used for uncoupled expression of *arsR^R773^*. **Fig. S3.** Relevant construction details of the feedback (A) and uncoupled (B) circuits. Sequences show part of the *arsR* gene, the various promoters, the ArsR Binding Sites (ABS) and the start of the *egfp* reporter gene. **Fig. S4.** Arsenite-dependent EGFP fluorescence in cultures of *E. coli* MG1655 with different uncoupled *arsR* reporter circuits (pAAUN, pLtetOUN, pJJUN, pVUN, pKUN) compared with the feedback-controlled *arsR-egfp* circuit on pPR-arsR-ABS-egfp. NFU, culture density normalized fluorescence after 120 min induction time using fluorimeter measurements. Data symbols represent the average from independent biological triplicates. Whiskers, SD (when not visible lay within the symbol size). **Fig. S5.** Time response kinetics of the EGFP fluorescence signal in *E. coli* MG1655 carrying the different feedback and uncoupled bioreporter circuits, at different arsenite concentrations between 0 and 20 μg l^−1^ and measured in fluorimetry. NFU, culture density normalized fluorescence. Data points show triplicate averages ± one SD.

**SI File 1.** Excel version of the ArsR-P_ars_ models in feedback or uncoupled modes.
