# Supplementary material for: Tunable reporter signal production in feedback-uncoupled arsenic bioreporters
Source: Microb Biotechnol. 2013 Jan 15;6(5):503–14. doi: 10.1111/1751-7915.12031 (PMC3918153; doi:10.1111/1751-7915.12031)
Supplement: Supplementary file 1 — Table S1. List of all the primers used in the present work showing sequence, length and melting temperature (Tm). Fig. S1. Nucleotide alignment of the arsRR73 and the chromosomal arsRK12 genes. Fig. S2. Relevant part of the DNA sequence of the different promoters used for uncoupled expression of arsRR773. Fig. S3. Relevant construction details of the feedback (A) and uncoupled (B) circuits. Sequences show part of the arsR gene, the various promoters, the ArsR Binding Sites (ABS) and the start of the egfp reporter gene. Fig. S4. Arsenite-dependent EGFP fluorescence in cultures of E. coli MG1655 with different uncoupled arsR reporter circuits (pAAUN, pLtetOUN, pJJUN, pVUN, pKUN) compared with the feedback-controlled arsR-egfp circuit on pPR-arsR-ABS-egfp. NFU, culture density normalized fluorescence after 120 min induction time using fluorimeter measurements. Data symbols represent the average from independent biological triplicates. Whiskers, SD (when not visible lay within the symbol size). Fig. S5. Time response kinetics of the EGFP fluorescence signal in E. coli MG1655 carrying the different feedback and uncoupled bioreporter circuits, at different arsenite concentrations between 0 and 20 μg l−1 and measured in fluorimetry. NFU, culture density normalized fluorescence. Data points show triplicate averages ± one SD. [file mbt0006-0503-sd1.docx]

- Supplementary information -

**Tunable reporter signal production in feedback-uncoupled arsenic bioreporters**

Davide Merulla^1^, Vassily Hatzimanikatis^2,3^, Jan Roelof van der Meer^1,^*

1) Department of Fundamental Microbiology, University of Lausanne, 1015 Lausanne

Switzerland.

2) Laboratory of Computational Systems Biotechnology, Ecole Polytechnique Fédérale de Lausane (EPFL), CH 1015 Lausanne, Switzerland. 3) Swiss Institute of Bioinformatics (SIB), CH 1015 Lausanne, Switzerland.

- Mathematical model

- Table S1

- Figures S1-S5

**Mathematical model for ArsR- circuits.**

**Symbols**

m_A_ mRNA of *arsR* (dimensionless)

M_A_ *arsR* mRNA concentration (M)

G genome concentration, 1 molecule/cell ~4·10^-9^ (M)

*G* is used to scale to dimensionless concentrations, that correspond to relative copy numbers per cell.

$$m_{A}=\frac{M_{A}}{G}$$

m_F_ mRNA of *gfp* (dimensionless)

M_F_ *gfp* mRNA concentration (M)

$$m_{F}=\frac{M_{F}}{G}$$

ρ_A_ ArsR protein (dimensionless)

P_A_ ArsR protein concentration (M)

$$\rho_{A}=\frac{P_{A}}{G}$$

ρ_F_ GFP protein (dimensionless)

P_F_ GFP protein concentration (M)

$$\rho_{F}=\frac{P_{F}}{G}$$

$\frac{g_{P}}{G}$ plasmid copies per cell. Value: 10

$\bar{K}_{mA}=\frac{K_{mA}}{K_{d,mA}}$ copies M_A_ from DNA per G. Value: 8.

$\bar{K}_{mAp}=\frac{K_{mAp}}{K_{d,mA}}$ copies M_A_ from plasmid per G. Value: 8.

$\bar{K}_{mF}=\frac{K_{mF}}{K_{d,mF}}$ copies M_F_ from plasmid per G. Value: 12.

Assumption 1: transcription efficiency same for chromosome and plasmid DNA.

$\varphi=\frac{K_{d,mF}}{K_{d,mA}}$ =$\frac{t_{1/2,MA}}{t_{1/2,MF}}$ ratio of mRNA half-lives. Value: 1.

$\frac{1}{\lambda}\cong\frac{K_{mA}}{K_{pA}}\times\frac{K_{dpA}}{K_{dmA}}$ = $\frac{mRNA copies}{protein copies}$

λ_A_ ArsR protein copies per *arsR* mRNA. Value: 5.

λ_F_ GFP protein copies per *gfp* mRNA. Value: 5.

$h_{A}=\frac{K_{dpA}}{K_{dmA}}$ $=\frac{t_{1/2,MA}}{t_{1/2,PA}}$

equivalent to the ratio of *arsR* mRNA half-life over ArsR protein half-life.

Value *h*_A_: 0.5.

K_A_ equilibrium constant for ArsR binding to its DNA binding site.

Value K_A_: 2·10^12^ (M^-1^).

K_C_ equilibrium constant for binding of AsIII to ArsR.

Value K_C_: 1·10^7·n^ (M^-n^)

n number of molecules AsIII bound per molecule of ArsR. Value: 2.

K_D_ equilibrium constant for ArsR-As binding to its DNA binding site.

Value K_D_: 2·10^9^ (M^-1^)

ε concentration of AsIII (M)

η_D_ efficiency of transcription from P_ars_

η_E_ efficiency of transcription from P_x_, one of the constitutive promoters

**Case A: *arsR* and *gfp* under control of Pars. No secondary binding site. Only plasmid copies.**

$$\frac{dm_{A}}{d\tau} = \bar{K}_{m_{A}}\cdot\eta_{p}(\rho_{A})\cdot\frac{g_{p}}{G} - m_{A}$$

$$\frac{dm_{F}}{d\tau} = \bar{K}_{m_{F}}\cdot\eta_{p}(\rho_{A})\cdot\varphi\cdot\frac{g_{p}}{G} - {\varphi\cdot m}_{F}$$

$$\frac{d\rho_{A}}{d\tau} = \bar{K}_{m_{A}}\cdot\lambda_{A}\cdot h_{A}\cdot m_{A}-h_{A}\cdot\rho_{A}$$

$$\frac{d\rho_{F}}{d\tau} = \bar{K}_{m_{F}}\cdot\lambda_{F}\cdot\varphi\cdot h_{F}\cdot m_{F}-h_{F}\cdot\varphi\cdot\rho_{F}$$

in steady state:

$$\frac{dm_{A}}{d\tau} = 0 and \frac{d\rho_{A}}{d\tau} = 0$$

thus:

$$m_{A}= \bar{K}_{m_{A}}\cdot\eta_{p}(\rho_{A})\cdot\frac{g_{p}}{G}$$

and:

$$h_{A}\cdot\rho_{A}= \bar{K}_{m_{A}}\cdot\lambda_{A}\cdot h_{A}\cdot m_{A} , in other words: \rho_{A}= \bar{K}_{m_{A}}\cdot\lambda_{A}\cdot m_{A}$$

with:

$\eta_{p}= \frac{1}{\alpha_{D}\cdot\rho_{A}+1}$

and:

$\alpha_{D}= \frac{K_{A}+K_{C}\cdot K_{D}\cdot\epsilon^{n}}{1+K_{C}\cdot\epsilon^{n}}\cdot G$

substitute $m_{A}$, then

$$\rho_{A}= (\bar{K}_{m_{A}})^{2}\cdot\eta_{p}\cdot\frac{g_{p}}{G}\cdot\lambda_{A} = \frac{(\bar{K}_{m_{A}})^{2}\cdot\frac{g_{p}}{G}\cdot\lambda_{A}}{\alpha_{D}\cdot\rho_{A}+1} = \frac{A}{\alpha_{D}\cdot\rho_{A}+1}$$

with $A=(\bar{K}_{m_{A}})^{2}\cdot\frac{g_{p}}{G}\cdot\lambda_{A}$

then:

$$\alpha_{D}\cdot{(\rho}_{A})^{2}+\rho_{A}= A$$

solve:

Will give two solutions of type a = b ± c. Only the expression with '+' makes biologically sense.

$$\rho_{A}= - \frac{1}{{2\alpha}_{D}}+ \sqrt{\left( \frac{1}{{2\alpha}_{D}} \right)^{2}+\frac{A}{\alpha_{D}}}$$

similarly, in steady state:

$$\frac{dm_{F}}{d\tau} = 0 and \frac{d\rho_{F}}{d\tau} = 0$$

then:

$${\varphi\cdot m}_{F}= \bar{K}_{m_{F}}\cdot\eta_{p}(\rho_{A})\cdot\varphi\cdot\frac{g_{p}}{G} , in other words: m_{F}= \bar{K}_{m_{F}}\cdot\eta_{p}(\rho_{A})\cdot\frac{g_{p}}{G}$$

and

$$h_{F}\cdot\varphi\cdot\rho_{F}= \bar{K}_{m_{F}}\cdot\lambda_{F}\cdot\varphi\cdot h_{F}\cdot m_{F} , in other words: \rho_{F}= \bar{K}_{m_{F}}\cdot\lambda_{F}\cdot m_{F}$$

substitute $m_{F}$

$$\rho_{F}= \frac{(\bar{K}_{m_{F}})^{2}\cdot\frac{g_{p}}{G}\cdot\lambda_{F}}{\alpha_{D}\cdot\rho_{A}+1}$$

**Case B: *arsR* under Px and *gfp* under control of ArsR/Pars. No secondary binding site. Only plasmid copies.**

$$\frac{dm_{A}}{d\tau} = \bar{K}_{m_{A}}\cdot\eta_{E}\cdot\frac{g_{p}}{G} - m_{A}$$

$$\frac{dm_{F}}{d\tau} = \bar{K}_{m_{F}}\cdot\eta_{p}(\rho_{A})\cdot\varphi\cdot\frac{g_{p}}{G} - {\varphi\cdot m}_{F}$$

$$\frac{d\rho_{A}}{d\tau} = \bar{K}_{m_{A}}\cdot\lambda_{A}\cdot h_{A}\cdot m_{A}-h_{A}\cdot\rho_{A}$$

$$\frac{d\rho_{F}}{d\tau} = \bar{K}_{m_{F}}\cdot\lambda_{F}\cdot\varphi\cdot h_{F}\cdot m_{F}-h_{F}\cdot\varphi\cdot\rho_{F}$$

Constant ArsR:

$$\frac{dm_{A}}{d\tau} = 0 and \frac{d\rho_{A}}{d\tau} = 0$$

thus:

$$h_{A}\cdot\rho_{A}= \bar{K}_{m_{A}}\cdot\lambda_{A}\cdot h_{A}\cdot m_{A} , in other words: m_{A}= \frac{h_{A}\cdot\rho_{A}}{\bar{K}_{m_{A}}\cdot\lambda_{A}\cdot h_{A}} = \frac{\rho_{A}}{\bar{K}_{m_{A}}\cdot\lambda_{A}}$$

then:

$$0= \bar{K}_{m_{A}}\cdot\eta_{E}\cdot\frac{g_{p}}{G}- \frac{\rho_{A}}{\bar{K}_{m_{A}}\cdot\lambda_{A}}$$

thus:

$$\frac{\rho_{A}}{\bar{K}_{m_{A}}\cdot\lambda_{A}}= \bar{K}_{m_{A}}\cdot\eta_{E}\cdot\frac{g_{p}}{G} or: \rho_{A}=\lambda_{A}\cdot\left( \bar{K}_{m_{A}} \right)^{2}\cdot\eta_{E}\cdot\frac{g_{p}}{G}$$

similarly, in steady state:

$$\frac{dm_{F}}{d\tau} = 0 and \frac{d\rho_{F}}{d\tau} = 0$$

then:

$${\varphi\cdot m}_{F}= \bar{K}_{m_{F}}\cdot\eta_{p}(\rho_{A})\cdot\varphi\cdot\frac{g_{p}}{G} , in other words: m_{F}= \bar{K}_{m_{F}}\cdot\eta_{p}(\rho_{A})\cdot\frac{g_{p}}{G}$$

and

$$h_{F}\cdot\varphi\cdot\rho_{F}= \bar{K}_{m_{F}}\cdot\lambda_{F}\cdot\varphi\cdot h_{F}\cdot m_{F} , in other words: \rho_{F}= \bar{K}_{m_{F}}\cdot\lambda_{F}\cdot m_{F}$$

substitute $m_{F}$

$$\rho_{F}= \frac{\left( \bar{K}_{m_{F}} \right)^{2}\cdot\frac{g_{p}}{G}\cdot\lambda_{F}}{\alpha_{D}\cdot\rho_{A}+1}$$

**Case A2: *arsR* and *gfp* under control of Pars. No secondary binding site. Chromosome *arsR* and plasmid copies.**

$$\frac{dm_{A}}{d\tau} = \bar{K}_{m_{A}}\cdot\eta_{D}(\rho_{A})+\bar{K}_{m_{A}p}\cdot\eta_{p}(\rho_{A})\cdot\frac{g_{p}}{G} - m_{A}$$

$$\frac{dm_{F}}{d\tau} = \bar{K}_{m_{F}}\cdot\eta_{p}(\rho_{A})\cdot\varphi\cdot\frac{g_{p}}{G} - {\varphi\cdot m}_{F}$$

$$\frac{d\rho_{A}}{d\tau} = \bar{K}_{m_{A}}\cdot\lambda_{A}\cdot h_{A}\cdot m_{A}-h_{A}\cdot\rho_{A}$$

$$\frac{d\rho_{F}}{d\tau} = \bar{K}_{m_{F}}\cdot\lambda_{F}\cdot\varphi\cdot h_{F}\cdot m_{F}-h_{F}\cdot\varphi\cdot\rho_{F}$$

under steady state:

$$\frac{dm_{A}}{d\tau} = 0 \mathrm{and} \frac{d\rho_{A}}{d\tau} = 0$$

thus:

$$m_{A}= \bar{K}_{m_{A}}\cdot\eta_{D}(\rho_{A})+\bar{K}_{m_{A}}\cdot\eta_{p}(\rho_{A})\cdot\frac{g_{p}}{G}$$

and:

$$h_{A}\cdot\rho_{A}= \bar{K}_{m_{A}}\cdot\lambda_{A}\cdot h_{A}\cdot m_{A} , in other words: \rho_{A}= \bar{K}_{m_{A}}\cdot\lambda_{A}\cdot m_{A}$$

with:

$\eta_{D}= \frac{1}{\alpha_{D}\cdot\rho_{A}+1}$

assume: η_D_ = η_p_

and:

$\alpha_{D}= \frac{K_{A}+K_{C}\cdot K_{D}\cdot\epsilon^{n}}{1+K_{C}\cdot\epsilon^{n}}\cdot G$

substitute $m_{A}$, then

$$\rho_{A}= (\bar{K}_{m_{A}})^{2}\cdot\eta_{D}\cdot\lambda_{A}+ (\bar{K}_{m_{A}})^{2}\cdot\eta_{D}\cdot\frac{g_{p}}{G}\cdot\lambda_{A} = \frac{\lambda_{A}\cdot(\bar{K}_{m_{A}})^{2}\cdot\left( 1+\frac{g_{p}}{G} \right)}{\alpha_{D}\cdot\rho_{A}+1} = \frac{A}{\alpha_{D}\cdot\rho_{A}+1}$$

with $A=\lambda_{A}\cdot(\bar{K}_{m_{A}})^{2}\cdot\left( 1+\frac{g_{p}}{G} \right)$

then:

$$\alpha_{D}\cdot{(\rho}_{A})^{2}+\rho_{A}= A$$

solve (similar remark on the two possible solutions; only '+' makes biologically sense)

$$\rho_{A}= - \frac{1}{{2\alpha}_{D}}+ \sqrt{\left( \frac{1}{{2\alpha}_{D}} \right)^{2}+\frac{A}{\alpha_{D}}}$$

similarly, in stead state:

$$\frac{dm_{F}}{d\tau} = 0 and \frac{d\rho_{F}}{d\tau} = 0$$

then:

$${\varphi\cdot m}_{F}= \bar{K}_{m_{F}}\cdot\eta_{p}(\rho_{A})\cdot\varphi\cdot\frac{g_{p}}{G} , in other words: m_{F}= \bar{K}_{m_{F}}\cdot\eta_{p}(\rho_{A})\cdot\frac{g_{p}}{G}$$

and

$$h_{F}\cdot\varphi\cdot\rho_{F}= \bar{K}_{m_{F}}\cdot\lambda_{F}\cdot\varphi\cdot h_{F}\cdot m_{F} , in other words: \rho_{F}= \bar{K}_{m_{F}}\cdot\lambda_{F}\cdot m_{F}$$

substitute $m_{F}$

$$\rho_{F}= \frac{(\bar{K}_{m_{F}})^{2}\cdot\frac{g_{p}}{G}\cdot\lambda_{F}}{\alpha_{D}\cdot\rho_{A}+1}$$

**Case B2: *arsR* under Px and *gfp* under control of ArsR/Pars on plasmid. No secondary binding site. Additional chromosomal *arsR* copy.**

$$\frac{dm_{A}}{d\tau} = \bar{K}_{m_{A}}\cdot\eta_{D}(\rho_{A})+\bar{K}_{m_{Ap}}\cdot\eta_{E}\cdot\frac{g_{p}}{G} - m_{A}$$

$$\frac{dm_{F}}{d\tau} = \bar{K}_{m_{F}}\cdot\eta_{p}(\rho_{A})\cdot\varphi\cdot\frac{g_{p}}{G} - {\varphi\cdot m}_{F}$$

$$\frac{d\rho_{A}}{d\tau} = \bar{K}_{m_{A}}\cdot\lambda_{A}\cdot h_{A}\cdot m_{A}-h_{A}\cdot\rho_{A}$$

$$\frac{d\rho_{F}}{d\tau} = \bar{K}_{m_{F}}\cdot\lambda_{F}\cdot\varphi\cdot h_{F}\cdot m_{F}-h_{F}\cdot\varphi\cdot\rho_{F}$$

Under steady state:

$$\frac{dm_{A}}{d\tau} = 0 and \frac{d\rho_{A}}{d\tau} = 0$$

then:

$$h_{A}\cdot\rho_{A}= \bar{K}_{m_{A}}\cdot\lambda_{A}\cdot h_{A}\cdot m_{A} , in other words: m_{A}= \frac{h_{A}\cdot\rho_{A}}{\bar{K}_{m_{A}}\cdot\lambda_{A}\cdot h_{A}} = \frac{\rho_{A}}{\bar{K}_{m_{A}}\cdot\lambda_{A}}$$

substitute:

$$0= \bar{K}_{m_{A}}\cdot\eta_{D}(\rho_{A})+\bar{K}_{m_{A}p}\cdot\eta_{E}\cdot\frac{g_{p}}{G}- \frac{\rho_{A}}{\bar{K}_{m_{A}}\cdot\lambda_{A}}$$

thus:

$$\frac{\rho_{A}}{\bar{K}_{m_{A}}\cdot\lambda_{A}}= \bar{K}_{m_{A}}\cdot\eta_{D}(\rho_{A})+\bar{K}_{m_{A}p}\cdot\eta_{E}\cdot\frac{g_{p}}{G}$$

with:

$\eta_{D}= \frac{1}{\alpha_{D}\cdot\rho_{A}+1}$

and:

$\alpha_{D}= \frac{K_{A}+K_{C}\cdot K_{D}\cdot\epsilon^{n}}{1+K_{C}\cdot\epsilon^{n}}\cdot G$

substitute:

$$\rho_{A}= \lambda_{A}\cdot\bar{K}_{m_{A}}\cdot\bar{K}_{m_{A}p}\cdot\eta_{E}\cdot\frac{g_{p}}{G}+ \frac{\lambda_{A}\cdot\left( \bar{K}_{m_{A}} \right)^{2}}{\alpha_{D}\cdot\rho_{A}+1}$$

substitute:

$$A= \lambda_{A}\cdot\left( \bar{K}_{m_{A}} \right)^{2}$$

and

$$B= \lambda_{A}\cdot\bar{K}_{m_{A}}\cdot\bar{K}_{m_{A}p}\cdot\eta_{E}\cdot\frac{g_{p}}{G}$$

solve:

$$\rho_{A}= -\left( \frac{1-\alpha_{D}\cdot B}{2\alpha_{D}} \right)+\sqrt{\left( \frac{1-\alpha_{D}\cdot B}{2\alpha_{D}} \right)^{2}+\left( \frac{A+B}{\alpha_{D}} \right)}$$

similarly, in steady state

$$\frac{dm_{F}}{d\tau} = 0 and \frac{d\rho_{F}}{d\tau} = 0$$

then:

$${\varphi\cdot m}_{F}= \bar{K}_{m_{F}}\cdot\eta_{p}(\rho_{A})\cdot\varphi\cdot\frac{g_{p}}{G} , in other words: m_{F}= \bar{K}_{m_{F}}\cdot\eta_{p}(\rho_{A})\cdot\frac{g_{p}}{G}$$

and

$$h_{F}\cdot\varphi\cdot\rho_{F}= \bar{K}_{m_{F}}\cdot\lambda_{F}\cdot\varphi\cdot h_{F}\cdot m_{F} , in other words: \rho_{F}= \bar{K}_{m_{F}}\cdot\lambda_{F}\cdot m_{F}$$

substitute $m_{F}$

$$\rho_{F}= \frac{\left( \bar{K}_{m_{F}} \right)^{2}\cdot\frac{g_{p}}{G}\cdot\lambda_{F}}{\alpha_{D}\cdot\rho_{A}+1}$$

**Supplementary Table S1**. List of all the primers used in the present work showing sequence, length and melting temperature (Tm).

| **Primer code** | **Function** | **Length** | **Calculated Tm (°C)** | **Sequence 5'-3'** |
| --- | --- | --- | --- | --- |
| 010907 | Seq. constitutive promoter | 29 | 59.4 | AATTCACATAACCAAAAACGCATATGATG |
| 110711 | Seq. constitutive promoter | 29 | 61.6 | CGCACAACTCTCCCATCTCCCTG |
| 090722 | Seq. coupled sys. mCherry insertion | 23 | 60.8 | TAACCTTCGGGCATGGCACTCTT |
| 070817 | Seq. uncoupled sys. mCherry insertion | 24 | 65 | CTGCCAGGAATTGGGGATCGGAAG |
| 100107 | Chromosomal K.O. | 27 | 59.9 | GAATTCTTGGTATGGACGAAATGTTGC |
| 100108 | Chromosomal K.O. | 26 | 61.9 | ACTAGTCGCTTCTGACATATTGCGCTCCTG |
| 100109 | Chromosomal K.O. | 30 | 65.2 | GAATTCCTTTGAAAGCGTTTATGCGC |
| 100110 | Chromosomal K.O. | 30 | 62.0 | ACTAGTCGCTTCAGTAACATAATGCCTCCC |
| 100111 | Chromosomal K.O. | 27 | 60.8 | GAAGCGACTAGTCGCCTGAAATAAAGC |
| 100112 | Chromosomal K.O. | 30 | 59.2 | GGGATCCCATATTGATCAGAGATATATCCT |

**Supplementary Figure S1.** Nucleotide alignment of the *arsR^R73^* and the chromosomal *arsR^K12^* genes.

**Figure S2.** Relevant part of the DNA sequence of the different promoters used for uncoupled expression of *arsR^R773^*.

**Figure S3**. Relevant construction details of the feedback (A) and uncoupled (B) circuits. Sequences show part of the *arsR* gene, the various promoters, the ArsR Binding Sites (ABS) and the start of the *egfp* reporter gene.

**Figure S4.** Arsenite-dependent EGFP fluorescence in cultures of *E. coli* MG1655 with different uncoupled *arsR*-reporter circuits (pAAUN, pLtetOUN, pJJUN, pVUN, pKUN) compared to the feedback-controlled *arsR*-*egfp* circuit on pPR-arsR-ABS-egfp. NFU, culture density normalized fluorescence after 120 min induction time using fluorimeter measurements. Data symbols represent the average from independent biological triplicates. Whiskers, *SD* (when not visible lay within the symbol size).

**Figure S5.** Time response kinetics of the EGFP fluorescence signal in *E. coli* MG1655 carrying the different feedback and uncoupled bioreporter circuits, at different arsenite concentrations between 0 and 20 µg/L and measured in fluorimetry. NFU, culture density normalized fluorescence. Data points show triplicate averages ± one *SD.*
